# Diversity and environmental adaptation of phagocytic cell metabolism

**DOI:** 10.1002/JLB.4RI0518-195R

**Published:** 2018-09-14

**Authors:** Luke C. Davies, Christopher M. Rice, Daniel W. McVicar, Jonathan M. Weiss

**Affiliations:** ^1^ Cancer & Inflammation Program National Cancer Institute Frederick Maryland USA; ^2^ Division of Infection & Immunity School of Medicine Cardiff University Heath Park UK

**Keywords:** glycolysis, niche diversity, oxidative phosphorylation, phagocyte metabolism

## Abstract

Phagocytes are cells of the immune system that play important roles in phagocytosis, respiratory burst and degranulation—key components of innate immunity and response to infection. This diverse group of cells includes monocytes, macrophages, dendritic cells, neutrophils, eosinophils, and basophils—heterogeneous cell populations possessing cell and tissue‐specific functions of which cellular metabolism comprises a critical underpinning. Core functions of phagocytic cells are diverse and sensitive to alterations in environmental‐ and tissue‐specific nutrients and growth factors. As phagocytic cells adapt to these extracellular cues, cellular processes are altered and may contribute to pathogenesis. The considerable degree of functional heterogeneity among monocyte, neutrophil, and other phagocytic cell populations necessitates diverse metabolism. As we review our current understanding of metabolism in phagocytic cells, gaps are focused on to highlight the need for additional studies that hopefully enable improved cell‐based strategies for counteracting cancer and other diseases.

## 1 FUNCTIONAL DIVERSITY IN PHAGOCYTIC CELLS

Phagocytic cells, or phagocytes, were first clearly described by Ilya Metchnikoff as microphages (neutrophils) and macrophages inside inflamed tissues.^1^ Phagocytes consume large particles through phagocytosis, which differs from the more common pinocytosis used to uptake molecules.^2^ Neutrophils and monocytes/ macrophages make up a major part of innate immunity, and are required for the phagocytic clearance of pathogens, a theory originally suggested by Ilya Metchnikoff.^3^ Both neutrophils and macrophages can be derived from bone marrow precursors, though it is now well appreciated that a large number of macrophage populations are independently derived from yolk sac or fetal liver precursors, and maintain their populations through local proliferation.^4^ Regardless of origin, all phagocytes share not only their engulfing function, but they also share downstream mechanisms, such as phagolysosome formation and respiratory burst.^5^ Nonetheless, phagocyte diversity exists because of unique functions. This is evident in inflammation, where tissue‐resident macrophages recruit neutrophils, which subsequently recruit monocytes that differentiate into inflammatory macrophages that are eventually cleared by the returning tissue‐resident macrophages. Each phagocyte performs specific functions that cannot be completely compensated for by other phagocytes.^6^ Additionally, macrophages can suppress neutrophil functions^7^ and tissue‐resident macrophages can suppress infiltrating monocyte‐derived macrophage phagocytosis^8^ to control inflammation for preservation of tissue integrity and limit auto‐immunity. We here review how recent findings have enhanced our understanding of how myeloid cell subsets meet specific metabolic demands in disease.

### 1.1 Metabolism underpins myeloid cell function

**Figure 1:**
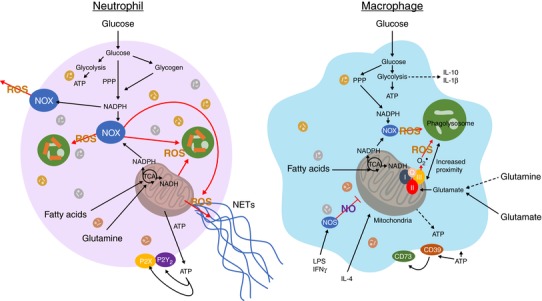
**Metabolic pathways of neutrophils and macrophages**. Macrophages and neutrophils utilize their differing metabolic capacities to maintain their effector function. In short lived, rapidly responding neutrophils, glucose is rapidly processed to form ATP via glycolysis and NADPH via the pentose phosphate pathway. These activities enable neutrophils to engage in chemotaxis, phagocytose and maintain respiratory burst via NADPH. Neutrophil NOX can be associated with the plasma membrane, rather than the mitochondria. This glucose‐maintained NADPH source is important for many ROS related functions such as NET formation; however fatty acid and glutamine fueled mitochondrial function has also been demonstrated to play a significant role in NET formation and the maintenance of ROS when glucose availability or NOX function is limited. Neutrophils also build and utilize glycogen, possibly to further aid function in glucose‐depleted environments. Macrophages are also able to process glucose via glycolysis and the pentose phosphate pathway to maintain ATP and NADPH respectively. ATP‐stimulation of macrophages helps maintain mitochondrial membrane potential, cell viability and induces cytokine expression. As a longer‐lived cell, macrophages also contain significant mitochondrial capacity and utilize this to process glutamine and fatty acids. This mitochondrial capacity is enhanced during activation with IL‐4 and reduced under IFN‐γ and LPS stimulation in an NO‐dependent mechanism

Metabolism is the process whereby cells convert fuel and food into energy and the building blocks of life. One of the first major findings in the field of cell metabolism occurred when Lois Pasteur determined that bad batches of wine in France were caused by the production of lactic acid from sugar.^9^ Fifty years later, it was discovered that pyruvate formed lactic acid under homeostatic conditions in animals,^10^ and that lactic acid was produced by muscles, under anaerobic conditions.^11^ Otto Warburg showed that tumor cells could produce lactic acid aerobically, which was later referred to as the Warburg effect.^12^ Eventually, these observations and others led to the discovery of parallel pathways whereby glucose is oxidized, either by the glycolytic pathway whereby pyruvate and energy in the form of ATP and reduced NADH is formed,^13^ or via the pentose phosphate pathway (PPP), which yields the formation of NADPH and nucleotide precursors such as ribose 5‐phosphate. Subsequently, Hans Krebs and Arthur Johnson determined that pyruvate fed into the TCA cycle for ATP production,^14^ a pathway involving oxidative phosphorylation (OXPHOS), which is a contributor of energetic metabolism and formation of reactive oxygen species (ROS), such as superoxide and hydrogen peroxide. Phagocytic cells, when properly stimulated, utilize metabolic pathways via a process referred to as respiratory burst to generate ROS necessary for pathogen killing (Table 1). Glycolytically derived ATP can have autocrine effects on activated macrophages, such as the maintenance of mitochondrial membrane potential, protection from apoptotic cell death, and production of chemokines that are in turn important for neutrophil recruitment^15^, ^16^ (Fig. 1).

**Table 1:** Metabolic pathways in phagocytic cell subsets. The table denotes metabolic pathways utilized by specific phagocytic cells for cellular functions. (ROS, reactive oxygen species; FAO, fatty acid oxidation; FAS, fatty acid synthesis; TAM, tumor associated macrophage; CARKL, carbohydrate kinase‐like protein; NET, neutrophil extracellular traps)

	Glycolysis	PPP	OXPHOS/ ETC	TCA cycle	Fatty acids	Amino acids
BMDM + LPS/IFN‐γ	Enhanced: Survival and Cytokines^28^	Enhanced: ROS, NO, Redox, RNA^34^	Shut down via NO^27^ and itaconic acid^112^	Broken^38^: Itaconic acid, Lipids, Cytokines^28^	Enhanced FAO & FAS: Cytokines^135^, ^136^	Glutamine: Not needed for phenotype^38^ Arginine: NO production^27^
BMDM + IL‐4	Enhanced: Phenotype maintenance^35^	Shut down via CARKL: Phenotype maintenance^34^	Enhanced: ATP, Phenotype maintenance^33^		Enhanced FAO & FAS: Phenotype maintenance^33^, ^36^	Glutamine: protein modifications maintain phenotype^38^ Arginine: Polyamines, Proline for proliferation/ repair^137^, ^138^
cDC + LPS	Enhanced: Survival^26^ and Cytokines^24^	Enhanced: ROS, NO, Redox, RNA^139^	Shut down via NO^26^	Enhanced: Lipid production^139^	Enhanced FAS: Phenotype maintenance^139^	Arginine: NO production^26^
pDC + CpG	Delayed enhancement: Cytokine production^140^		Enhanced: Cytokine production^140^		Enhanced FAS & FAO: Cytokine production^140^	
Peritoneal ResMΦ (+phagocytosis)	Enhanced: ATP production^64^	Enhanced: ROS production^64^	Enhanced: Phagocytosis, ROS production, microbial killing^64^	Complex II enhanced: ROS production^64^	Enhanced FAO after Il‐4: Phenotype, proliferation^36^	Glutamine & Glutamate: ROS production. Basal arginase expression^64^
Neutrophils/ gMDSC	Enhanced: ATP production^64^	Enhanced: ROS production^64^ NET formation^94^	NET formation, ROS^97^ ATP production^90^		Autophagic FAO: Differentiation^87^ NADPH production for ROS T‐cell Suppressive function (Rice 2018)^120^	Glutamine not required for phagocytosis/ respiratory burst^64^ Contributes to NET formation^96^
TAM/mMDSCs	Lactate stabilized HIF1a in TAMs^106^ ‐ induced glycolysis?				T‐cell Suppressive function	Arginine (via arginase) and Tryptophan (via IDO): T cell suppression in tumor^141^, ^142^, ^143^, ^144^

### 1.2 Macrophage and dendritic cell metabolism

Years after the discovery of the Warburg effect, it was appreciated that peripheral blood neutrophils and monocytes primarily undergo lactic acid producing glycolysis, whereas alveolar macrophages utilize glycolysis for mitochondrial fueling.^17^, ^18^ In addition to aerobic glycolysis, neutrophils also use the pentose phosphate pathway after phagocytosis,^19^ to produce free radicals utilized in the killing of microbes, a mechanism that is also recognized in macrophages. After these early findings, the field of phagocyte immunology grew and interest in metabolic function was superseded by integral discoveries of novel subsets and functions. We are only now beginning to assess the impact of Warburg metabolism in specific cell subsets.^20^, ^21^, ^22^, ^23^ In 2010, it was reported that dendritic cells (DCs) enhance their levels of glycolysis upon sensing of Toll‐like receptor ligands (Table 1).^24^ This study, supported by insights from lymphocyte immunometabolism,^25^ triggered a dramatic reevaluation of how metabolism supports phagocyte function. This enhancement of glycolysis was attributed to a compensation effect for NO‐mediated inhibition of OXPHOS,^26^ a phenomenon additionally reported in macrophages.^27^ Importantly, blocking glycolysis in these macrophages specifically inhibited their ability to produce the cytokines IL‐1β^28^ and IL‐10 (Fig. 1).^27^ In the case of IL‐10, both macrophage and DC autocrine signaling restricts the up‐regulation of glycolysis,^24^, ^27^ and suppresses NO production, limiting subsequent reductions in OXPHOS (Table 1).^27^ Additionally, IL‐10 can promote mitophagy, in the absence of this signal the inflammasome becomes activated by damaged mitochondrial components and IL‐1β is released, promoting tissue inflammation.^29^

Although it is known that glycolysis increases within minutes of LPS addition,^27^ the mechanisms that control this immediate impact are incompletely understood. However, it has been widely reported that hypoxia inducible factor 1α (HIF1α) can be stabilized after LPS sensing in macrophages and maintains glycolysis over the long‐term by enhancing glycolytic gene expression.^28^, ^30^ HIF1α can work in concert with an isozyme of pyruvate kinase (PKM2).^31^ Normally inactive, dimeric/monomeric PKM2 translocates to the nucleus and is involved in the activation of HIF1α target genes, reportedly via a kinase activity rather than its enzyme function, although this remains controversial.^31^ PKM2 is not the only metabolic enzyme to exhibit moonlighting activity: aconitase is also known as iron regulatory protein 1 and can control the translation of mRNAs containing iron‐responsive elements and GAPDH can bind AU‐rich elements of mRNA, such as IFN‐γ. GAPDH is also part of the IFN‐γ‐activated inhibitor of translation (GAIT) complex that controls inflammatory gene transcription.^32^ More studies are required to determine the repertoire of metabolic enzyme moonlighting and how this affects phagocyte functions; these will involve unraveling the integration of cell function with metabolic demands.

Mitochondrial OXPHOS is required for alternative activation of macrophages with IL‐4.^33^ IL‐4 triggers Stat6/proliferator‐activated receptor‐γ (PPAR‐γ) mediated changes that suppress the pentose phosphate pathway via CARKL,^34^ elevate glycolysis,^35^ and enhances OXPHOS through increased lipid breakdown and fatty acid oxidation^33^, ^36^ (Fig. 1 and Table 1). These metabolic processes in resting and IL‐4 treated cells are reportedly maintained by CSF‐1 or IL‐4 signaling through mTORC2 and IRF4.^35^ Chronic parasitic diseases may also elicit tissue‐specific changes in metabolic programming. Schistosome worm survival and egg production are highly dependent on OXPHOS and primarily use fatty acids acquired from their hosts to fuel OXPHOS via beta‐oxidation.^37^ The general enhancement in metabolism, along with suppression of the pentose phosphate pathway (that supports inflammatory activation via NADPH oxidase [NOX]) seen after IL‐4 is expected considering the enhanced metabolic demand and reported anti‐inflammatory properties of IL‐4‐stimulated macrophages (Fig. 1). However, this emphasizes the metabolic switch seen with inflammatory activation, where there is increased demand, but lower mitochondrial function.

The suppression of OXPHOS in LPS‐activated macrophages could occur through multiple mechanisms, including the down‐regulation of Idh1,^38^ deactivation of pyruvate dehydrogenase,^39^ and IFN‐regulated gene 1 (Irg1)‐mediated itaconate suppression of mitochondrial complex II.^40^ Suppression of OXPHOS is primarily attributable to NO, as deletion of inducible NOS2 completely restored OXPHOS.^27^ NO reportedly disrupts iron‐sulfur centers in metabolic enzymes such as cytosolic aconitase,^41^ and can specifically inhibit cytochrome C oxidase.^42^ In contrast, alternative activation of macrophages via IL‐4 has been linked to a substantial up‐regulation of the metabolic enzyme arginase (Table 1). This is important because arginase and NOS can compete for their substrate, arginine. Arginase has a much lower binding affinity for arginine than NOS, but in resting or IL‐4 stimulated macrophages its expression is greatly larger than NOS. However, activation with LPS results in enhanced NOS expression, which can now effectively compete with arginase and produce NO.^43^, ^44^ It is unknown whether this suppression of OXPHOS is an off‐target effect of an antimicrobial agent or is actively required for inflammatory macrophage function. It has been argued that the shutdown of OXPHOS is required for citrate accumulation, a major source of extramitochondrial acetyl‐CoA (via ATP‐citrate lyase), which can fuel lipid production or diffuse through the nuclear membrane for histone acetylation.^45^ Interestingly, THP‐1 monocytes were shown to uptake extracellular citrate for maintenance of histone acetylation and enhanced production of pro‐inflammatory cytokines.^46^ Therefore, citrate may be an interesting metabolic target; however Pdk1 suppression of pyruvate entry into the TCA in activated macrophages seems to oppose citrate production, demonstrating that more study is required to dissect these complex mechanisms.

Although it is appreciated that OXPHOS is suppressed 18–24 h after LPS (Table 1), this is not indicative of the early response to bacterial components. A study by Garaude et al.^47^ reports that bone marrow‐derived macrophages alter their super‐complex (quaternary combinations of mitochondrial complexes to allow efficient electron transfer) structures after sensing live bacteria to favor enhanced complex II activity and produce ROS that is required for optimal bacterial killing. Additionally, Mills et al.^48^ report that complex II‐dependent ROS is also required for inflammasome activation and production of IL‐1β. However, it is still unclear how these metabolic changes in macrophages translate into in vivo metabolic environments and whether a therapeutic window can be achieved.

In summary, metabolic differences between classically and alternatively activated macrophages clearly demonstrate the importance and linkage between metabolism and cellular function.^49^ Increased glycolytic flux in M1 macrophages maintains ATP levels for biosynthesis, favors NADPH production by the pentose phosphate pathway, and results in NO and ROS generation (Fig. 1). Conversely, M2 macrophages generate ATP primarily through OXPHOS and fatty acid oxidation, which can be sustained for longer periods of time. Macrophages modulate their metabolic pathways due to alterations in their environment and thus their cellular phenotype is sensitive to metabolic adaptations.

### 1.3 Unique characteristics of tissue‐resident macrophage metabolism

Now considered distinct from traditional monocyte‐derived macrophages, tissue‐resident macrophages, in the majority of tissues can be derived from yolk sac or fetal liver precursors, and maintain their populations through local proliferation.^4^ However, monocytes can eventually replenish some of these populations to become long‐lived cells,^50^ and gut macrophages are constantly renewed by monocytes.^51^ The full impact of a macrophage's origin on function has yet to be elucidated; however it is known that monocyte‐derived cells are less resistant to radiation induced DNA damage than nonmyeloid‐derived macrophages such as Langerhans cells.^52^ Regardless of cellular origin, the mechanisms that govern macrophage differentiation and adaptation to specific niches will facilitate understanding of tissue‐specific physiology, which is distinct from studying these cells after long term in vitro culture.

Tissue‐resident macrophages play important roles in tissue and organismal metabolism. This is evident in metabolic organs such as the liver, where Kupffer cells engage in iron metabolic homeostasis^53^ and fat storage in adipose tissue.^54^ In the context of cellular energetic metabolism, however, very little has been recorded in tissue‐resident macrophages. An intricate understanding of cell metabolic crosstalk in privileged sites such as the brain has emerged. Examples include the mechanisms by which astrocytes, part of the blood brain barrier, convert blood sugar into lactate, a usable fuel for neurons, and the continual uptake and recycling of neuronal secreted neurotransmitters N‐acetyl‐aspartate (NAA) and glutamate by glial cells.^55^, ^56^ In the latter case, the oligodendrocytes specifically express the aspartoacylase enzyme required for NAA breakdown. The catabolism of NAA is needed to restrict the neurotransmitter level, but it is additionally vital for oligodendrocyte production of the sphingomyelin that is required for effective myelination of the neurons; impairment of aspartoacylase results in Canavan's disease.^57^ Although these interactions are intriguing, the relevance of these intercellular metabolic connections in tissues readily accessible to blood nutrients remains poorly understood.

Recent studies in the peritoneal cavity have further enhanced our view on how cells metabolically adapt to maintain tissue homeostasis. The peritoneum is a serosal nexus of well‐vascularized distinctive organs, and the professional tissue‐resident macrophages here can invade adjacent organs to facilitate tissue repair.^58^ Peritoneal macrophages have a unique gene signature controlled by epigenetic alterations brought about by the peritoneal environment.^59^, ^60^ One such factor is the metabolite retinoic acid, which is manufactured by omental tissue from vitamin A.^61^ Retinoic acid acts through retinoid X receptor (RXR) receptors to activate Gata6 expression, which is a master controller of the peritoneal macrophage phenotype.^61^, ^62^, ^63^ This not only has functional contexts for the cells, but Gata6 expression is also required for maintenance of peritoneal B1 cells; through transforming growth factor β2 secretion, these B1‐cells participate in gut IgA production, making this an important pathway for immune protection of the organism.^61^ A recent study by our laboratory showed that the peritoneal cavity also contains NAA,^64^ which is noteworthy because Gata6‐driven aspartoacylase is also expressed in peritoneal tissue‐resident macrophages.^62^ It is likely that the peritoneal tissue‐resident macrophages play a role in clearance of peritoneal NAA, but more study is required to elucidate the role of NAA in the peritoneum. Additionally, our study^64^ highlighted that the peritoneum contains glutamate, which can be utilized by peritoneal tissue‐resident macrophages during phagocytosis to fuel mitochondrial function for enhanced ROS production (Table 1). This mechanism is dependent on protein kinase C with subsequent ROS production almost completely dependent on mitochondrial complex III activity. Thus, peritoneal macrophages are unlikely to rely on reverse electron transfer for their considerable ROS production, as has been reported with bone marrow‐derived macrophages. Additionally, unlike bone marrow‐derived macrophages,^47^ peritoneal macrophages are poised to utilize complex II without the additional need for microbial sensing mechanisms. These differences inform us that the metabolic set up of various cell subsets in situ is likely to differ from studies in vitro, and care needs to be taken in the interpretation of these results. The source of peritoneal glutamate and its effects on other peritoneal cells are still unknown, though it is well placed to engage glutamate receptors, perhaps on parasympathetic neurons; meaning that macrophage metabolic demands can alter the physiology of the peritoneal tissue.

Peritoneal macrophages are dependent on CSF‐1, which acts as a survival and/or growth factor,^65^ and it has been reported that bone marrow‐derived macrophages also utilize CSF‐1 signaling to maintain metabolic pathways via mTORC2 and IRF‐4.^35^ However, a recent study^66^ reveals that these mechanisms can become complex in the peritoneal environment. Retinoic acid‐driven Gata6 expression maintains the peritoneal macrophage phenotype, but also competes with mTORC2, which can down‐regulate Gata6 expression via phosphorylation of FOXO1. This inhibits proliferation and survival, and is seemingly an oxymoron, as CSF‐1 is required for survival, but could actively signal mTORC2 to restrict survival. It is likely that Gata6 and CSF‐1 are both acting to keep the cell relatively quiescent, but this is modified during inflammation, so that mTORC2 can repurpose the metabolic programming to facilitate inflammatory function, although this may result in the death of some cells, as has been theorized in the macrophage disappearance reaction.^67^ Another example of the importance of cell energetics in tissue‐resident macrophages comes from a recent study by Ulland et al.^68^ TREM2 signaling was required to activate mTOR and AMP‐activated protein kinase (AMPK) to manage the increased energetic demands of microglia in brain inflammation. In this case, the authors fixed the microglial energy deficit through dectin‐1 signaling or in vivo cyclocreatine addition, this reduced aberrant autophagy and reduced neuronal damage. This study shows that understanding both macrophage functions and metabolic demands can lead to metabolically targeted treatment strategies.

These studies on tissue‐resident macrophages emphasize the need for investigating phagocyte functions in context with their tissue niche environment. Although studies on isolated cells are extremely useful in interrogating cell signaling pathways, they may ultimately constrain our understanding of how tissue systems work in concert to maintain or restore homeostasis, which in turn will hinder our ability to treat disease. It is expected that additional integrative approaches to studying cell interactions with the environment will combine our current understanding of phagocyte function with tissue physiology.

### 1.4 Neutrophils

Neutrophils are a specialized subset of phagocytes, which are abundant, short lived, highly motile, and aggressively defend against pathogens. Neutrophils represent the most numerous leukocyte in human blood with a high turnover rate with as much as 60% of the bone marrow committed to their development^69^ and as many at 10^9^/kg released from the bone marrow each day and a further 6 × 10^11^ prepared for release.^70^ Although neutrophils were thought to survive only hours in the circulation,^71^ recent studies have shown they can last days in circulation^72^ and subsequently their roles in homeostasis^73^ and diseases such as cancer^74^ are being reevaluated. Neutrophils have very low transcriptional and translational activity^75^ and have the majority of proteins they require for function preassembled and packaged in cytoplasmic granules.^76^ This preparation allows neutrophils to instantly respond to activating signals without the need to engage in transcription and protein translation. However, this lack of cellular activity and short life span has led to a similar assumption of neutrophil metabolism, as neutrophils, for the most part, can be assumed to not require the complex metabolic networks that other cells use to maintain life spans and engage in long‐term functions. Hence, an oversimplification that has plagued them in the wider immunology field has continued into immunometabolism, leaving neutrophils often overlooked as a metabolically simple cell, wholly dependent on glycolysis.

These initial assumptions of glucose dependence have some merit and are based on early observations of phagocyte metabolism. For example, neutrophils contain far fewer number of active mitochondrial compared to other professional phagocytes such as macrophages.^77^, ^78^ In addition to this, studies have reported that, as part of their maturation process, neutrophils lose cytochrome C expression,^79^ a vital component of the electron transport chain and maintain mitochondrial membrane potential to engage in apoptosis.^79^ Furthermore, neutrophil effector functions were shown to be highly dependent on glucose. Upon activation, neutrophils engage in the generation of large amounts of ROS, via respiratory burst. Thus, neutrophils, like monocytes and macrophages, use the NOX system^80^ which uses molecular oxygen and NADPH to produce superoxide (Fig. 1). This is in turn processed into other downstream ROS by the action of enzymes such as myeloperoxidase. This activity is essential for neutrophil function and competent protection against microbes. To this end neutrophils engage their propensity for glucose metabolism to fuel the pentose phosphate pathway and glucose‐6‐phosphate (G6P) dehydrogenase, which catalyzes the reaction of G6P to 6‐phospho‐ gluconolactone, yielding NADPH. Patients with mutations in this enzyme suffer from G6P dehydrogenase deficiency (G6PDD), which in severe forms of this mutation can lead to reduced NADPH levels required to effectively run the NOX system, leading reduced neutrophil ROS and increased susceptibility to infection.^81^, ^82^ Additionally, mutations that affect neutrophil handling of the glucose derivative, G6P, via the G6P transporter leads to a severe congenital neutropenia.^83^ This can lead to arrest in neutrophil development and dysfunction of respiratory burst and chemotaxis.^84^ This loss of G6P/glucose homeostasis in these neutrophils leads to reduced glucose uptake, impaired lactate, ATP and NADPH production, activation of HIF‐1α/PPAR‐γ pathways, and cellular dysfunction.^82^ Neutrophils have also been shown to store the glucose polymer glycogen at the inflammatory site, perhaps suggesting that in some circumstances neutrophils store glucose for later use (Fig. 1).^85^ These studies underline the importance of glucose in neutrophil metabolism and its effects on effector function.

Neutrophil mitochondria have also been shown to play a role in important cellular functions. Despite initial observations of mitochondrial loss during development, recent studies have demonstrated that mitochondrial function^86^ and a switch from glycolysis to fatty acid oxidation is required for effective neutrophil differentiation in vivo (Fig. 1).^87^ Furthermore, other studies have now suggested active networks of mitochondria are present in circulating neutrophils and are required for responses such as chemotaxis, through release of low‐level cell signaling ROS.^88^ Although disruption of mitochondrial function was not shown to affect respiratory burst or phagocytosis initially, prolonged disruption of mitochondrial membrane potential inhibited respiratory burst, suggesting a steady state role for mitochondrial metabolism in neutrophils.^89^ Additionally, mitochondrial‐dependent autocrine purinergic signaling is important for neutrophil activation. Despite requiring glycolysis to engage in prolonged signaling, mitochondria appear to be essential in initiating this signaling response.^90^

Mitochondria can also act as an ROS‐producing organelle.^91^ This mechanism has been shown to play a role in host defense from pathogens by macrophages.^47^, ^92^ This is mechanism is particularly interesting when considering patients with chronic granulomatous disease (CGD). CGD is a heterogeneous disease leading to defects in the ROS‐producing NOX system. This lack of phagocyte ROS leaves patients highly susceptible to pathogens. Interestingly, studies in both mice and humans demonstrated that treatment with the PPAR‐γ agonist pioglitazone enhanced mitochondrial ROS in NOX‐deficient neutrophils. This induced mitochondrial ROS and partially restored the ability of NOX‐deficient neutrophils to combat staphylococcus aureus, demonstrating a potential compensatory mechanism of mitochondria to support NOX activity.^93^ Neutrophil extracellular traps (NETs) are of recent interest to the field of neutrophil metabolism. These structures consist of DNA and granules and are used to trap and kill invading pathogens, a process termed NETosis. NETosis requires ROS production, and as described above, involves a metabolic shift toward PPP and G6PD activity to fuel NOX activity (Fig. 1).^94^ Indeed, G6PDD leads to a reduced ability of neutrophils to produce NETs.^95^ Further studies have revealed that mitochondrial function also plays a role in NET formation (Table 1). Although still largely dependent on increased glucose uptake and metabolism, withdrawal of glutamine or disruption of mitochondrial ATPase was also shown to partially reduce NET formation.^96^ This suggested a glutamine‐fueled mitochondrial‐dependent stage to NETosis in neutrophils. Indeed, during the engagement of NETosis, neutrophil mitochondria translocate to the cell surface and become hyperpolarized releasing ROS.^97^ In addition to mitochondrial ROS involvement, mitochondrial components, such as oxidized mitochondrial DNA, have also been shown to be a component of NETs.^98^

Despite the above studies, we still know very little of how neutrophils adapt their metabolism to function in the environment around them and whether this can be altered in disease. Recent data from our laboratory has shown that neutrophil mitochondrial metabolism supports respiratory burst by fueling NOX with NADPH.^99^ One important distinction between neutrophils and macrophages is the plasma membrane association of NOX in neutrophils (Fig. 1).^100^ Thus, plasma membrane associated NOX in neutrophils is not coupled to mitochondrial metabolism. Neutrophils that possess a relatively greater mitochondrial mass, such as immature neutrophils, maintain NADPH production via fatty acid‐dependent mitochondrial function when glucose metabolism was restricted.^99^ This effect was particularly prominent in tumor‐elicited neutrophils, which had significantly enhanced mitochondrial mass, function, and associated fatty acid metabolism. Neutrophils require fatty acid oxidation and mitochondrial respiration for differentiation from the bone marrow^87^ and immature neutrophilic cell subsets are recruited to tumors.^101^ In consideration of this, we hypothesize that like macrophages,^64^ neutrophil mitochondria may contribute to respiratory burst in glucose‐deprived environments such the tumor microenvironment. This could be either by direct ROS contribution^93^ or citrate export from the mitochondria and subsequent IDH1 activity to supply NADPH fuel for NOX. Concordantly, immature neutrophils would have a functional advantage, as they are no longer wholly dependent on glucose metabolism to maintain effector functions. Because low‐density, possibly immature, neutrophils more readily engage in mitochondria‐dependent NET formation,^102^ the heterogeneity in neutrophil morphology, surface marker expression and metabolism may be a dictating factor of neutrophil functional outcome. It is important to consider that heterogeneous neutrophil subsets may be metabolically adapted into differing tissue niches, especially during chronic inflammation. As numerous neutrophil subsets are being discovered and better characterized in both steady state and disease,^103^ it is intriguing to hypothesize that these subsets are targetable due to their differing metabolic architecture.

### 1.5 Adaptations of phagocyte metabolism in cancer

As mentioned, phagocytic cells adapt to specific microenvironments in their utilization of fuels to derive cellular metabolism. Moreover, all cells are sensitive to alterations in their microenvironment; thus it is clear that metabolic alterations in immune cells underlie a central component of pathology. In cancer, the competitive advantage for tumor cells to consume nutrients such as glucose has the profound potential to limit availability of fuels necessary for immune cell function.^104^ Although the metabolism of tumor cells has been well described, it is noteworthy that the effects of tumor cells upon the immunometabolism of macrophages and other immune cells are incompletely understood. Small amounts of extracellular citrate can be incorporated into tumor cells at physiologic levels found in the blood, and this is increased in hypoxic glucose‐starved conditions that match the tumor setting.^105^ As mentioned previously, activated macrophages accumulate citrate^38^; thus, it would be interesting to hypothesize that macrophage activation in the tumor setting can supply extracellular citrate for tumor progression. Additionally, the high levels of glycolysis in tumors yield lactic acid, which may polarize tumor‐associated macrophages into a pro‐tumor, M2‐like phenotype.^106^ There are presumably many undiscovered fuels and signals associated with tumors involved in metabolic alterations and polarization in macrophages. Other mechanisms by which tumor‐associated macrophage differentiation may be facilitated are highlighted by the caspase‐dependent cleavage of peroxisome PPAR‐γ, which results in the inhibition of fatty acid oxidation and accumulation of tumor associated macrophage (TAM)‐promoting lipid droplets^107^ Another intriguing mechanism by which tumors may influence phagocyte metabolism is by alterations in expression or function by cell surface proteins, such as HSP90.^108^ Although HSP90 is a typical cytoplasmic/nuclear chaperone protein capable of regulating mitophogy and cytokine production in macrophages, it also comprises a pool of cell surface associated proteins potentially regulated by extracellular cues such as tumors. In DCs, the TLR‐mediated detection of danger signals and subsequent cellular responses to pathogens is in part through alterations in mitochondrial metabolism.^24^, ^26^ The tissue‐specific composition and availability of fuels also underlies niche‐specific alterations in metabolism.^64^ Wenes et al. show that tumor‐associated macrophages contribute to abnormal blood vessel formation and angiogenesis, through the utilization of large amounts of glucose and inhibition of mTOR that promotes quiescence of vascular endothelial cells.^109^ In that study, the hypoxia‐inducible mTOR inhibitor, REDD1 plays an important role in regulating glucose utilization by tumor‐associated macrophages and may be an attractive metabolic target, because REDD1‐targeting resulted in normalization of tumor vessels and reduced metastases. Tissues such as the peritoneal cavity are frequently the site of tumor development and metastasis, and as such there is considerable potential crosstalk between developing tumors and tissue‐resident macrophages that normally play important roles in homeostasis and immune surveillance. We recently showed that crosstalk between tumor cells and resident macrophages of the peritoneum induces metabolic alterations in the resident macrophage population, which in turn regulates macrophage effector function and tumor progression.^110^ In ovarian carcinoma, which metastasizes to and grows progressively in the peritoneum, altered metabolite expression is highlighted by the dramatic up‐regulation of the metabolite, itaconic acid, in resident macrophages.^110^ Itaconic acid was already recognized as an important metabolite due to its ability to inhibit the glyoxylate shunt, an anabolic variation of the TCA that is essential for bacterial growth.^111^ In mammalian cells, itaconic acid inhibits glycolysis and the TCA, the latter by acting on succinate dehydrogenase.^112^, ^113^ Itaconic acid is robustly induced by proinflammatory stimuli such as IFN‐γ or LPS.^114^ In our studies, itaconic acid was a critical component of the tumor‐induced enhancement of fatty acid oxidation‐fueled OXPHOS in resident macrophages and the generation of pro‐tumor ROS.^110^ The identification of metabolites such as itaconic acid and REDD1 as potential therapeutic targets supports the need for additional research into the multitude of ways tumor and host cellular responses are regulated by metabolism.

### 1.6 Myeloid derived suppressor cells

Myeloid derived suppressor cells (MDSC) are defined as a heterogeneous population of phagocytes that inhibit antitumor immunity through various mechanisms.^115^ In mice, these cells are defined by Gr‐1 positive staining. This has led to extreme confusion as Gr‐1 identifies both Ly‐6G and Ly‐6C expressed primarily on neutrophils and monocytes respectively. This lack of differentiation makes the reported studies hard to interpret, particularly from a metabolic standpoint. Some studies have further subdivided the Gr‐1 positive populations using Ly6G and Ly6C to identify the granulocytic (g)‐MDSC and monocytic (m)‐MDSC respectively.^116^ Difference between these MDSC subsets and classically identified neutrophils and monocytes remain ill‐defined and may indeed represent an activated state of these subsets. Human MDSC remain even harder to define as there is no equivalent to Gr‐1, which detects both monocytic and granulocytic cells together.

Regardless of their identity, studies have identified metabolic underpinning to the differentiation, maintenance, and suppressive nature of these heterogeneous phagocytic populations. In vitro differentiation of BM precursors to MDSC‐like phenotype using GM‐CSF and IL‐6 enhanced glucose uptake and anaerobic glycolysis as well as glutamine utilization.^117^ Furthermore, recent studies have demonstrated that tumors increased expression of genes involved in glycolysis in both g‐ and m‐MDSC subsets.^118^ This increased glycolysis was required to promote expansion and prevent apoptosis of MDSC.

Fatty acid metabolism has also been shown to be a vital component of MDSC development and function (Table 1). Conditioned media and diets high in poly‐unsaturated fatty acids have been shown to promote development of g‐MDSC subsets in vitro and in vivo.^119^ Hossain et al. demonstrated that MDSC populations increase fatty acid uptake, mitochondrial mass, and oxygen consumption rates at the tumor site.^120^ This fatty acid‐dependent metabolic function was found to be required for their suppressive function and cytokine production. Interestingly this study suggested that fatty acid uptake was increased in both Ly‐6G and Ly‐6C positive cells at the tumor site, suggesting that several subsets of activated phagocyte may become more oxidative in the tumor microenvironment.

MDSC have also shown to alter the metabolic milieu of the environment around them to fulfill their suppressive function. Many of these studies highlight the ability of MDSC to deplete the local niche of amino acids, required for T cell activity. T cells require the amino acid cysteine for protein synthesis and activation following antigen presentation. However, T cells struggle to acquire this metabolite themselves due to the lack enzyme systems required to form cysteine or the transporters that import the oxidized analogue cystine from the extracellular environment. T cells are therefore wholly dependent on the activity of supporting phagocytes, such as DCs and macrophages, which create cysteine by reducing imported cystine or by converting methionine to cysteine by the action of cystathionase. These phagocytes then export cysteine, which is utilized by the T cells during activation.^121^ MDSC disrupt this metabolic corporation by consuming extracellular cystine and failing to generate and export cysteine.^122^ Therefore, the accumulation of MDSC depletes the extracellular environment of cysteine and renders T cells inactive. MDSC can also engage in heightened arginine metabolism to modify a tumor milieu. This occurs by enhanced arginase‐1 activity, which depletes extra cellular arginine, rendering T cell inactive through down regulation of TCR zeta (Table 1). This mechanism was originally described in macrophages,^123^ suggesting that many aspects of MDSC metabolic activity are not unique to this population. In addition to its ability to deplete arginine, the activity of the arginase was found to be essential in controlling the glucose and glutamine uptake as well as TCA activity and subsequent AMP‐kinase activity of in vitro MDSC models.^117^ MDSC have been shown to deplete extracellular tryptophan by the action of the enzyme IDO, further contributing to an immune suppressive tumor niche.^124^ MDSC also generate reactive free radicals such as ROS and NO, which have detrimental effects on T cell activity. The MDSC in a similar manner to neutrophils and macrophages produce large quantities of ROS vis NOX.^125^, ^126^ Whereas, g‐MDSC appear to be a greater source of arginase activity in tumors, the metabolism of L‐arginine to citrullene and NO by NOS is a metabolic pathway more associated with the m‐MDSC.^127^, ^128^ This production of NO is also inhibitory to T cell activity.^129^

### 1.7 Metabolism of other phagocytes

As our understanding of phagocyte metabolism continues to develop, it will be intriguing to consider the metabolism of other less well‐studied phagocytes, such as osteoclasts, eosinophils, mast cells, and basophils. Osteoclasts, in a similar manner to resident macrophages, possess a high degree of metabolic plasticity, with a high glycolytic and mitochondrial capacity, that enables them to carry out effector function in diverse and often hostile environments.^130^ With numerous bone pathologies associated with aberrant osteoclast activity, further understanding of their metabolism may lead to novel therapeutic strategies for diseases such as osteoporosis. Similar to neutrophils, other granulocytes such as eosinophils and mast cells also have a high propensity for glucose metabolism and the pentose phosphate pathway,^131^ with mast cells also engaging in glycogen metabolism.^132^ However, mast cells were also found to utilize glucose metabolism to synthesize lipids and, more recently, OXPHOS has been demonstrated to be required for mast cell effector functions such as degranulation and cytokine production.^133^ Furthermore, abnormal tryptophan metabolism in eosinophils was found to be a critical component in eosinophilia‐myalgia syndrome,^134^ further suggesting that an improved understanding of granulocyte metabolism may potentially unveil pathologic drivers and potential drug targets.

## 2 CONCLUDING REMARKS

Metabolic adaptation to environmental cues is an intrinsic property of all cells. Mitochondrial function is essential for immune cell function and as such, cells must adapt to changes in resources in their surroundings. Immune cells exhibit niche‐specific alterations in their metabolism that are dependent on tissue‐specific concentrations of metabolic fuels such as glucose, glutamate, and glutamine that underlie distinctive tissue‐specific functions. For example, metabolic adaptation of tissue‐resident phagocytic cells is necessary for these cells to carry out cellular processes necessary for tissue homeostasis, response to pathogens, and localized inflammatory responses. As cellular environments change during pathogenesis, immune cells must adapt to alterations in the availability of nutrients and other environmental changes. The metabolic reprogramming of phagocytic cells during tumor development, for example, is a major contributing factor to altered and, in many cases, co‐opted, cellular function.

As we continue to learn more about metabolic programming in diverse immune subsets, the deployment of metabolic‐based strategies aimed at immune cell reprogramming becomes more of a feasible means by which cancer and other diseases may be treated. As such, there is a paucity of information on the metabolism of phagocytic cells such as eosinophils, basophils, and neutrophils. Moreover, the considerable degree of functional heterogeneity among monocyte, neutrophil, and other phagocytic cell populations indicates a spectrum of metabolic function exists in these cells. There is considerable need to refine our understanding of metabolic function in these cells so that metabolic‐based therapies become a feasible strategy to treat cancer and other diseases.

## References

[1] Metchnikoff E . Leçons sur la pathologie comparée de l'inflammation. Paris: Masson; 1892.

[2] Pratten MK , Lloyd JB . Pinocytosis and phagocytosis: the effect of size of a particulate substrate on its mode of capture by rat peritoneal macrophages cultured in vitro. Biochim Biophys Acta. 1986;881:307–313. 3008849 10.1016/0304-4165(86)90020-6

[3] Gordon S . Elie Metchnikoff: father of natural immunity. Eur J Immunol. 2008;38:3257–3264. 19039772 10.1002/eji.200838855

[4] Davies LC , Taylor PR . Tissue‐resident macrophages: then and now. Immunology. 2015;144:541–548. 25684236 10.1111/imm.12451 PMC4368161

[5] Dale DC , Boxer L , Liles WC . The phagocytes: neutrophils and monocytes. Blood. 2008;112:935–945. 18684880 10.1182/blood-2007-12-077917

[6] Soehnlein O , Lindbom L . Phagocyte partnership during the onset and resolution of inflammation. Nat Rev Immunol. 2010;10:427–439. 20498669 10.1038/nri2779

[7] Grainger JR , et al. Inflammatory monocytes regulate pathologic responses to commensals during acute gastrointestinal infection. Nat Med. 2013;19:713–721. 23708291 10.1038/nm.3189 PMC3755478

[8] Uderhardt S , et al. 12/15‐lipoxygenase orchestrates the clearance of apoptotic cells and maintains immunologic tolerance. Immunity. 2012;36:834–846. 22503541 10.1016/j.immuni.2012.03.010

[9] Pasteur L . Mèmoire sur la fermentation appeleé lactique. Annales de Chimie et de Physique. 1858;52:404–418.

[10] Mayer P . Über Brenztraubensäure‐Glucosurie und über das Verhalten der Brenztraubensäure im Tierkörper. Biochemische Zeitschrift. 1912;40:441.

[11] Meyerhof O . Über die Rolle der Milchsäure in der Energetik des Muskels. Naturwissenschaften. 1920;8:696–704.

[12] Warburg O , Posener K , Negelein E . Über den Stoffwechsel der Tumoren. Biochemische Zeitschrift. 1924;152:319–344.

[13] Kresge N , Simoni RD , Hill RL . Otto Fritz Meyerhof and the elucidation of the glycolytic pathway. J Biol Chem. 2005;280:e3. 15665335

[14] Krebs HA , Johnson WA . Metabolism of ketonic acids in animal tissues. Biochem J. 1937;31:645–660. 16746382 10.1042/bj0310645 PMC1266984

[15] Garedew A , Henderson SO , Moncada S . Activated macrophages utilize glycolytic ATP to maintain mitochondrial membrane potential and prevent apoptotic cell death. Cell Death Differ. 2010;17:1540–1550. 20339378 10.1038/cdd.2010.27

[16] Kawamura H , et al. Extracellular ATP‐stimulated macrophages produce macrophage inflammatory protein‐2 which is important for neutrophil migration. Immunology. 2012;136:448–458. 22564028 10.1111/j.1365-2567.2012.03601.x PMC3401983

[17] Beck WS . The control of leukocyte glycolysis. J Biol Chem. 1958;232:251–270. 13549415

[18] Oren R , et al. Metabolic patterns in three types of phagocytizing cells. J Cell Biol. 1963;17:487–501. 13940299 10.1083/jcb.17.3.487 PMC2106210

[19] Sbarra AJ , Karnovsky ML . The biochemical basis of phagocytosis. I. Metabolic changes during the ingestion of particles by polymorphonuclear leukocytes. J Biol Chem. 1959;234:1355–1362. 13654378

[20] O'Neill LA , Pearce EJ . Immunometabolism governs dendritic cell and macrophage function. J Exp Med. 2016;213:15–23. 26694970 10.1084/jem.20151570 PMC4710204

[21] Pearce EJ , Pearce EL . Driving immunity: all roads lead to metabolism. Nat Rev Immunol. 2017;18:81–82. 29226911 10.1038/nri.2017.139 PMC6312922

[22] O'Neill LAJ , Kishton RJ , Rathmell J . A guide to immunometabolism for immunologists. Nat Rev Immunol. 2016;16:553. 27396447 10.1038/nri.2016.70 PMC5001910

[23] Langston PK , Shibata M , Horng T . Metabolism supports macrophage activation. Front Immunol. 2017;8:1–7. 28197151 10.3389/fimmu.2017.00061 PMC5281575

[24] Krawczyk CM , et al. Toll‐like receptor‐induced changes in glycolytic metabolism regulate dendritic cell activation. Blood. 2010;115:4742–4749. 20351312 10.1182/blood-2009-10-249540 PMC2890190

[25] Pearce EL , et al. Fueling immunity: insights into metabolism and lymphocyte function. Science. 2013;342:1242454. 24115444 10.1126/science.1242454 PMC4486656

[26] Everts B , et al. Commitment to glycolysis sustains survival of NO‐producing inflammatory dendritic cells. Blood. 2012;120:1422–1431. 22786879 10.1182/blood-2012-03-419747 PMC3423780

[27] Baseler WA , et al. Autocrine IL‐10 functions as a rheostat for M1 macrophage glycolytic commitment by tuning nitric oxide production. Redox Biol. 2016;10:12–23. 27676159 10.1016/j.redox.2016.09.005 PMC5037266

[28] Tannahill GM , et al. Succinate is an inflammatory signal that induces IL‐1beta through HIF‐1alpha. Nature. 2013;496:238–242. 23535595 10.1038/nature11986 PMC4031686

[29] Ip WKE , et al. Anti‐inflammatory effect of IL‐10 mediated by metabolic reprogramming of macrophages. Science. 2017;356:513. 28473584 10.1126/science.aal3535 PMC6260791

[30] Cheng S‐C , et al. mTOR‐ and HIF‐1α–mediated aerobic glycolysis as metabolic basis for trained immunity. Science. 2014;345. 10.1126/science.1250684 PMC4226238 25258083

[31] Palsson‐McDermott EvaM , et al. Pyruvate kinase M2 regulates Hif‐1α activity and IL‐1β induction and is a critical determinant of the warburg effect in LPS‐activated macrophages. Cell Metab. 2015;21:65–80. 25565206 10.1016/j.cmet.2014.12.005 PMC5198835

[32] Castello A , Hentze MW , Preiss T . Metabolic enzymes enjoying new partnerships as RNA‐binding proteins. Trends Endocrinol Metab. 2015;26:746–757. 26520658 10.1016/j.tem.2015.09.012 PMC4671484

[33] Vats D , et al. Oxidative metabolism and PGC‐1beta attenuate macrophage‐mediated inflammation. Cell Metab. 2006;4:13–24. 16814729 10.1016/j.cmet.2006.05.011 PMC1904486

[34] Haschemi A , et al. The sedoheptulose kinase CARKL directs macrophage polarization through control of glucose metabolism. Cell Metab. 2012;15:813–826. 22682222 10.1016/j.cmet.2012.04.023 PMC3370649

[35] Huang SC , et al. Metabolic reprogramming mediated by the mTORC2‐IRF4 signaling axis is essential for macrophage alternative activation. Immunity. 2016;45:817–830. 27760338 10.1016/j.immuni.2016.09.016 PMC5535820

[36] Huang SC , et al. Cell‐intrinsic lysosomal lipolysis is essential for alternative activation of macrophages. Nat Immunol. 2014;15:846–855. 25086775 10.1038/ni.2956 PMC4139419

[37] Pearce EJ , Huang SC . The metabolic control of schistosome egg production. Cell Microbiol. 2015;17:796–801. 25850569 10.1111/cmi.12444 PMC4867551

[38] Jha AK , et al. Network integration of parallel metabolic and transcriptional data reveals metabolic modules that regulate macrophage polarization. Immunity. 2015;42:419–430. 25786174 10.1016/j.immuni.2015.02.005

[39] Tan Z , et al. Pyruvate dehydrogenase kinase 1 participates in macrophage polarization via regulating glucose metabolism. J Immunol. 2015;194:6082. 25964487 10.4049/jimmunol.1402469 PMC4458459

[40] Lampropoulou V , et al. Itaconate links inhibition of succinate dehydrogenase with macrophage metabolic remodeling and regulation of inflammation. Cell Metab. 2016;24:158–166. 27374498 10.1016/j.cmet.2016.06.004 PMC5108454

[41] Pearce LL , et al. The resistance of electron transport chain Fe‐S clusters to oxidative damage during the reaction of peroxynitrite with mitochondrial complex II and rat heart pericardium. Nitric Oxide: Biol Chem / Official Journal of the Nitric Oxide Society. 2009;20:135–142. 10.1016/j.niox.2008.12.001 PMC2692736 19118636

[42] Brown GC . Regulation of mitochondrial respiration by nitric oxide inhibition of cytochrome c oxidase. Biochim Biophys Acta (BBA) ‐ Bioenerg. 2001;1504:46–57. 10.1016/s0005-2728(00)00238-3 11239484

[43] Chang CI , Liao JC , Kuo L . Arginase modulates nitric oxide production in activated macrophages. Am J Physiol. 1998;274:H342–8. 9458885 10.1152/ajpheart.1998.274.1.H342

[44] Wu G . Arginine metabolism: nitric oxide and beyond. Biochem J. 1998;336:1–17. 9806879 10.1042/bj3360001 PMC1219836

[45] Wellen KE , et al. ATP‐citrate lyase links cellular metabolism to histone acetylation. Science. 2009;324:1076–1080. 19461003 10.1126/science.1164097 PMC2746744

[46] Ashbrook MJ , et al. Citrate modulates lipopolysaccharide‐induced monocyte inflammatory responses. Clin Exp Immunol. 2015;180:520–530. 25619261 10.1111/cei.12591 PMC4449780

[47] Garaude J , et al. Mitochondrial respiratory‐chain adaptations in macrophages contribute to antibacterial host defense. Nat Immunol. 2016;17:1037–1045. 27348412 10.1038/ni.3509 PMC4994870

[48] Mills EL , et al. Succinate dehydrogenase supports metabolic repurposing of mitochondria to drive inflammatory macrophages. Cell. 2016;167:457–470. e13. 27667687 10.1016/j.cell.2016.08.064 PMC5863951

[49] Galvan‐Pena S , O'Neill LA . Metabolic reprograming in macrophage polarization. Front Immunol. 2014;5:420. 25228902 10.3389/fimmu.2014.00420 PMC4151090

[50] Bain CC , et al. Long‐lived self‐renewing bone marrow‐derived macrophages displace embryo‐derived cells to inhabit adult serous cavities. Nat Commun. 2016;7:ncomms11852. 27292029 10.1038/ncomms11852 PMC4910019

[51] Bain CC , et al. Constant replenishment from circulating monocytes maintains the macrophage pool in the intestine of adult mice. Nat Immunol. 2014;15:929–937. 25151491 10.1038/ni.2967 PMC4169290

[52] Price JG , et al. The cell cycle inhibitor Cdkn1a regulates Langerhans cell radiation resistance and promotes T regulatory cell generation upon exposure to ionizing irradiation. Nat Immunol. 2015;16:1060–1068. 26343536 10.1038/ni.3270 PMC4620552

[53] Ganz T . Macrophages and systemic iron homeostasis. J Innate Immun. 2012;4:446–453. 22441209 10.1159/000336423 PMC6741611

[54] Mitro N , et al. The nuclear receptor LXR is a glucose sensor. Nature. 2006;445:219. 17187055 10.1038/nature05449

[55] Falkowska A , et al. Energy metabolism of the brain, including the cooperation between astrocytes and neurons, especially in the context of glycogen metabolism. Int J Mol Sci. 2015;16:25959–25981. 26528968 10.3390/ijms161125939 PMC4661798

[56] Amaral AI , et al. Metabolic aspects of neuron‐oligodendrocyte‐astrocyte interactions. Front Endocrinol (Lausanne). 2013;4:1–5. 23717302 10.3389/fendo.2013.00054 PMC3651962

[57] Namboodiri AMA , et al. Canavan disease and the role of N‐acetylaspartate in myelin synthesis. Mol Cell Endocrinol. 2006;252:216–223. 16647192 10.1016/j.mce.2006.03.016

[58] Wang J , Kubes P . A reservoir of mature cavity macrophages that can rapidly invade visceral organs to affect tissue repair. Cell. 2016;165:668–678. 27062926 10.1016/j.cell.2016.03.009

[59] Gosselin D , et al. Environment drives selection and function of enhancers controlling tissue‐specific macrophage identities. Cell. 2014;159:1327–1340. 25480297 10.1016/j.cell.2014.11.023 PMC4364385

[60] Lavin Y , et al. Tissue‐resident macrophage enhancer landscapes are shaped by the local microenvironment. Cell. 2014;159:1312–1326. 25480296 10.1016/j.cell.2014.11.018 PMC4437213

[61] Okabe Y , Medzhitov R . tissue‐specific signals control reversible program of localization and functional polarization of macrophages. Cell. 2014;157:832–844. 24792964 10.1016/j.cell.2014.04.016 PMC4137874

[62] Gautier EL , et al. Gata6 regulates aspartoacylase expression in resident peritoneal macrophages and controls their survival. J Exp Med. 2014;211:1525–1531. 25024137 10.1084/jem.20140570 PMC4113942

[63] Rosas M , et al. The transcription factor Gata6 links tissue macrophage phenotype and proliferative renewal. Science. 2014;344:645–648. 24762537 10.1126/science.1251414 PMC4185421

[64] Davies LC , et al. Peritoneal tissue‐resident macrophages are metabolically poised to engage microbes using tissue‐niche fuels. Nat Commun. 2017;8:2074. 29234000 10.1038/s41467-017-02092-0 PMC5727035

[65] Davies LC , et al. Distinct bone marrow‐derived and tissue resident macrophage‐lineages proliferate at key stages during inflammation. Nat Commun. 2013;4:1886. 23695680 10.1038/ncomms2877 PMC3842019

[66] Oh M‐H , et al. mTORC2 signaling selectively regulates the generation and function of tissue‐resident peritoneal macrophages. Cell Rep. 2017;20:2439–2454. 28877476 10.1016/j.celrep.2017.08.046 PMC5659290

[67] Barth MW , et al. Review of the macrophage disappearance reaction. J Leukoc Biol. 1995;57:361–367. 7884305 10.1002/jlb.57.3.361

[68] Ulland TK , et al. TREM2 maintains microglial metabolic fitness in Alzheimers disease. Cell. 2017;170:649–663. 28802038 10.1016/j.cell.2017.07.023 PMC5573224

[69] Kennedy AD , DeLeo FR . Neutrophil apoptosis and the resolution of infection. Immunol Res. 2009;43:25–61. 19066741 10.1007/s12026-008-8049-6

[70] Rankin SM . The bone marrow: a site of neutrophil clearance. J Leukoc Biol. 2010;88:241–251. 20483920 10.1189/jlb.0210112

[71] Cartwright GE , Athens JW , Wintrobe MM . The kinetics of granulopoiesis in normal man. Blood. 1964;24:780–803. 14235362

[72] Pillay J , et al. In vivo labeling with 2H2O reveals a human neutrophil lifespan of 5.4 days. Blood. 2010;116:625–627. 20410504 10.1182/blood-2010-01-259028

[73] Nicolas‐Avila JA , Adrover JM , Hidalgo A . Neutrophils in homeostasis, immunity, and cancer. Immunity. 2017;46:15–28. 28099862 10.1016/j.immuni.2016.12.012

[74] Coffelt SB , Wellenstein MD , de Visser KE . Neutrophils in cancer: neutral no more. Nat Rev Cancer. 2016;16:431–446. 27282249 10.1038/nrc.2016.52

[75] Geering B , Simon HU . Peculiarities of cell death mechanisms in neutrophils. Cell Death Differ. 2011;18:1457–1469. 21637292 10.1038/cdd.2011.75 PMC3178425

[76] Borregaard N , Sorensen OE , Theilgaard‐Monch K . Neutrophil granules: a library of innate immunity proteins. Trends Immunol. 2007;28:340–345. 17627888 10.1016/j.it.2007.06.002

[77] Chacko BK , et al. Methods for defining distinct bioenergetic profiles in platelets, lymphocytes, monocytes, and neutrophils, and the oxidative burst from human blood. Lab Invest. 2013;93:690–700. 23528848 10.1038/labinvest.2013.53 PMC3674307

[78] Kramer PA , et al. A review of the mitochondrial and glycolytic metabolism in human platelets and leukocytes: implications for their use as bioenergetic biomarkers. Redox Biol. 2014;2:206–210. 24494194 10.1016/j.redox.2013.12.026 PMC3909784

[79] Maianski NA , et al. Functional characterization of mitochondria in neutrophils: a role restricted to apoptosis. Cell Death Differ. 2004;11:143–153. 14576767 10.1038/sj.cdd.4401320

[80] Bedard K , Krause KH . The NOX family of ROS‐generating NADPH oxidases: physiology and pathophysiology. Physiol Rev. 2007;87:245–313. 17237347 10.1152/physrev.00044.2005

[81] Ardati KO , Bajakian KM , Tabbara KS . Effect of glucose‐6‐phosphate dehydrogenase deficiency on neutrophil function. Acta Haematol. 1997;97:211–215. 9158663 10.1159/000203685

[82] Jun HS , et al. Molecular mechanisms of neutrophil dysfunction in glycogen storage disease type Ib. Blood. 2014;123:2843–2853. 24565827 10.1182/blood-2013-05-502435 PMC4007611

[83] Boztug K , et al. A syndrome with congenital neutropenia and mutations in G6PC3. N Engl J Med. 2009;360:32–43. 19118303 10.1056/NEJMoa0805051 PMC2778311

[84] Chou JY , Jun HS , Mansfield BC . Neutropenia in type Ib glycogen storage disease. Curr Opin Hematol. 2010;17:36–42. 19741523 10.1097/MOH.0b013e328331df85 PMC3099242

[85] Robinson JM , Karnovsky ML , Karnovsky MJ . Glycogen accumulation in polymorphonuclear leukocytes, and other intracellular alterations that occur during inflammation. J Cell Biol. 1982;95:933–942. 7153252 10.1083/jcb.95.3.933 PMC2112917

[86] Six E , et al. AK2 deficiency compromises the mitochondrial energy metabolism required for differentiation of human neutrophil and lymphoid lineages. Cell Death Dis. 2015;6:e1856. 26270350 10.1038/cddis.2015.211 PMC4558504

[87] Riffelmacher T , et al. Autophagy‐dependent generation of free fatty acids is critical for normal neutrophil differentiation. Immunity. 2017;47:466–480. e5. 28916263 10.1016/j.immuni.2017.08.005 PMC5610174

[88] Vorobjeva N , et al. Mitochondrial reactive oxygen species are involved in chemoattractant‐induced oxidative burst and degranulation of human neutrophils in vitro. Eur J Cell Biol. 2017;96:254–265. 28325500 10.1016/j.ejcb.2017.03.003

[89] Fossati G , et al. The mitochondrial network of human neutrophils: role in chemotaxis, phagocytosis, respiratory burst activation, and commitment to apoptosis. J Immunol. 2003;170:1964–1972. 12574365 10.4049/jimmunol.170.4.1964

[90] Bao Y , et al. Mitochondria regulate neutrophil activation by generating ATP for autocrine purinergic signaling. J Biol Chem. 2014;289:26794–26803. 25104353 10.1074/jbc.M114.572495 PMC4175322

[91] Pinegin B , et al. The role of mitochondrial ROS in antibacterial immunity. J Cell Physiol. 2017. 10.1002/jcp.26117 28771715

[92] Davies LC , et al. Peritoneal tissue‐resident macrophages are metabolically poised to engage microbes using tissue‐niche fuels. Nat Commun. 2017;8:2074. 29234000 10.1038/s41467-017-02092-0 PMC5727035

[93] Fernandez‐Boyanapalli RF , et al. Pioglitazone restores phagocyte mitochondrial oxidants and bactericidal capacity in chronic granulomatous disease. J Allergy Clin Immunol. 2015;135:517–527. e12. 25498313 10.1016/j.jaci.2014.10.034 PMC4331116

[94] Azevedo EP , et al. A metabolic shift toward pentose phosphate pathway is necessary for amyloid Fibril‐ and phorbol 12‐myristate 13‐acetate‐induced neutrophil extracellular trap (NET) formation. J Biol Chem. 2015;290:22174–22183. 26198639 10.1074/jbc.M115.640094 PMC4571968

[95] Siler U , et al. Severe glucose‐6‐phosphate dehydrogenase deficiency leads to susceptibility to infection and absent NETosis. J Allergy Clin Immunol. 2017;139:212–219. e3. 27458052 10.1016/j.jaci.2016.04.041

[96] Rodriguez‐Espinosa O , et al. Metabolic requirements for neutrophil extracellular traps formation. Immunology. 2015;145:213–224. 25545227 10.1111/imm.12437 PMC4427386

[97] Lood C , et al. Neutrophil extracellular traps enriched in oxidized mitochondrial DNA are interferogenic and contribute to lupus‐like disease. Nat Med. 2016;22:146–153. 26779811 10.1038/nm.4027 PMC4742415

[98] Caielli S , et al. Oxidized mitochondrial nucleoids released by neutrophils drive type I interferon production in human lupus. J Exp Med. 2016;213:697–713. 27091841 10.1084/jem.20151876 PMC4854735

[99] Rice CM , DL , Subleski JJ , et al. Tumour‐elicited neutrophils engage mitochondrial metabolism to circumvent nutrient limitations and maintain immune inhibition. Nat Commun. 2018 **In Press**. 10.1038/s41467-018-07505-2 PMC6269473 30504842

[100] Shmelzer Z , et al. Unique targeting of cytosolic phospholipase A2 to plasma membranes mediated by the NADPH oxidase in phagocytes. J Cell Biol. 2003;162:683–692. 12913107 10.1083/jcb.200211056 PMC2173789

[101] Singel KL , Segal BH . Neutrophils in the tumor microenvironment: trying to heal the wound that cannot heal. Immunol Rev. 2016;273:329–343. 27558344 10.1111/imr.12459 PMC5477672

[102] Carmona‐Rivera C , Kaplan MJ . Low‐density granulocytes: a distinct class of neutrophils in systemic autoimmunity. Semin Immunopathol. 2013;35:455–463. 23553215 10.1007/s00281-013-0375-7 PMC4007274

[103] Garley M , Jablonska E . Heterogeneity among neutrophils. Arch Immunol Ther Exp (Warsz). 2018;66:21–30. 28560557 10.1007/s00005-017-0476-4 PMC5767199

[104] Chang CH , et al. Metabolic competition in the tumor microenvironment is a driver of cancer progression. Cell. 2015;162:1229–1241. 26321679 10.1016/j.cell.2015.08.016 PMC4864363

[105] Mycielska ME , et al. Extracellular citrate affects critical elements of cancer cell metabolism and supports cancer development in vivo. Cancer Res. 2018;78:2513–2523. 29510993 10.1158/0008-5472.CAN-17-2959

[106] Colegio OR , et al. Functional polarization of tumour‐associated macrophages by tumour‐derived lactic acid. Nature. 2014;513:559–563. 25043024 10.1038/nature13490 PMC4301845

[107] Niu Z , et al. Caspase‐1 cleaves PPARgamma for potentiating the pro‐tumor action of TAMs. Nat Commun. 2017;8:766. 28974683 10.1038/s41467-017-00523-6 PMC5626701

[108] Bzowska M , et al. Involvement of cell surface 90 kDa heat shock protein (HSP90) in pattern recognition by human monocyte‐derived macrophages. J Leukoc Biol. 2017;102:763–774. 28550115 10.1189/jlb.2MA0117-019R PMC5557637

[109] Wenes M , et al. Macrophage metabolism controls tumor blood vessel morphogenesis and metastasis. Cell Metab. 2016;24:701–715. 27773694 10.1016/j.cmet.2016.09.008

[110] Weiss JM , et al. Itaconic acid mediates crosstalk between macrophage metabolism and peritoneal tumors. J Clin Invest. 2018;128:3794–3805. 29920191 10.1172/JCI99169 PMC6118601

[111] Cordes T , Michelucci A , Hiller K . Itaconic Acid: the surprising role of an industrial compound as a mammalian antimicrobial metabolite. Annu Rev Nutr. 2015;35:451–473. 25974697 10.1146/annurev-nutr-071714-034243

[112] Cordes T , et al. Immunoresponsive gene 1 and itaconate inhibit succinate dehydrogenase to modulate intracellular succinate levels. J Biol Chem. 2016;291:14274–14284. 27189937 10.1074/jbc.M115.685792 PMC4933182

[113] Lampropoulou V , et al. Itaconate links inhibition of succinate dehydrogenase with macrophage metabolic remodeling and regulation of inflammation. Cell Metab. 2016;24:158–166. 27374498 10.1016/j.cmet.2016.06.004 PMC5108454

[114] Strelko CL , et al. Itaconic acid is a mammalian metabolite induced during macrophage activation. J Am Chem Soc. 2011;133:16386–16389. 21919507 10.1021/ja2070889 PMC3216473

[115] Gabrilovich DI , Nagaraj S . Myeloid‐derived suppressor cells as regulators of the immune system. Nat Rev Immunol. 2009;9:162–174. 19197294 10.1038/nri2506 PMC2828349

[116] Youn JI , et al. Subsets of myeloid‐derived suppressor cells in tumor‐bearing mice. J Immunol. 2008;181:5791–5802. 18832739 10.4049/jimmunol.181.8.5791 PMC2575748

[117] Hammami I , et al. Immunosuppressive activity enhances central carbon metabolism and bioenergetics in myeloid‐derived suppressor cells in vitro models. BMC Cell Biol. 2012;13:18. 22762146 10.1186/1471-2121-13-18 PMC3433355

[118] Jian SL , et al. Glycolysis regulates the expansion of myeloid‐derived suppressor cells in tumor‐bearing hosts through prevention of ROS‐mediated apoptosis. Cell Death Dis. 2017;8:e2779. 28492541 10.1038/cddis.2017.192 PMC5520713

[119] Yan D , et al. Polyunsaturated fatty acids promote the expansion of myeloid‐derived suppressor cells by activating the JAK/STAT3 pathway. Eur J Immunol. 2013;43:2943–2955. 23897117 10.1002/eji.201343472

[120] Hossain F , et al. Inhibition of fatty acid oxidation modulates immunosuppressive functions of myeloid‐derived suppressor cells and enhances cancer therapies. Cancer Immunol Res. 2015;3:1236–1247. 26025381 10.1158/2326-6066.CIR-15-0036 PMC4636942

[121] Gmunder H , et al., Macrophages regulate intracellular glutathione levels of lymphocytes. Evidence for an immunoregulatory role of cysteine. Cell Immunol. 1990;129:32–46. 2364441 10.1016/0008-8749(90)90184-s

[122] Srivastava MK , et al., Myeloid‐derived suppressor cells inhibit T‐cell activation by depleting cystine and cysteine. Cancer Res. 2010;70:68–77. 20028852 10.1158/0008-5472.CAN-09-2587 PMC2805057

[123] Rodriguez PC , et al., L‐arginine consumption by macrophages modulates the expression of CD3 zeta chain in T lymphocytes. J Immunol. 2003;171:1232–1239. 12874210 10.4049/jimmunol.171.3.1232

[124] Yu J. , et al., Myeloid‐derived suppressor cells suppress antitumor immune responses through IDO expression and correlate with lymph node metastasis in patients with breast cancer. J Immunol. 2013;190:3783–3797. 23440412 10.4049/jimmunol.1201449

[125] Corzo CA , et al., Mechanism regulating reactive oxygen species in tumor‐induced myeloid‐derived suppressor cells. J Immunol. 2009;182:5693–5701. 19380816 10.4049/jimmunol.0900092 PMC2833019

[126] Chen X , et al., Reactive oxygen species regulate T cell immune response in the tumor microenvironment. Oxid Med Cell Longev. 2016;2016:1580967. 27547291 10.1155/2016/1580967 PMC4980531

[127] Raber PL , et al., Subpopulations of myeloid‐derived suppressor cells impair T cell responses through independent nitric oxide‐related pathways. Int J Cancer. 2014;134:2853–2864. 24259296 10.1002/ijc.28622 PMC3980009

[128] Movahedi K. , et al., Identification of discrete tumor‐induced myeloid‐derived suppressor cell subpopulations with distinct T cell‐suppressive activity. Blood. 2008;111:4233–4244. 18272812 10.1182/blood-2007-07-099226

[129] Rossner S , et al., Myeloid dendritic cell precursors generated from bone marrow suppress T cell responses via cell contact and nitric oxide production in vitro. Eur J Immunol. 2005;35:3533–3544. 16331707 10.1002/eji.200526172

[130] Arnett TR , Orriss IR , Metabolic properties of the osteoclast. Bone. 2018;115:25–30. 29274806 10.1016/j.bone.2017.12.021

[131] DeChatelet LR , et al., Oxidative metabolism of the human eosinophil. Blood. 1977;50:525–535. 884325

[132] Chakravarty N , Glucose metabolism in rat mast cells. Stimulation of the pentose phosphate pathway by compound 48/80. Agents Actions. 1985;16:133–137. 3925733 10.1007/BF01983120

[133] Phong, B. , et al., Cutting edge: murine mast cells rapidly modulate metabolic pathways essential for distinct effector functions. J Immunol. 2017;198:640–644. 27974455 10.4049/jimmunol.1601150 PMC5225044

[134] Varga J , Jimenez SA , Uitto J , L‐tryptophan and the eosinophilia‐myalgia syndrome: current understanding of the etiology and pathogenesis. J Invest Dermatol. 1993;100:97S–105S. 8423409 10.1111/1523-1747.ep12356368

[135] Freigang S. , et al., Fatty acid‐induced mitochondrial uncoupling elicits inflammasome‐independent IL‐1alpha and sterile vascular inflammation in atherosclerosis. Nat Immunol. 2013;14:1045–1053. 23995233 10.1038/ni.2704

[136] Feingold KR , et al., Mechanisms of triglyceride accumulation in activated macrophages. J Leukoc Biol. 2012;92:829–839. 22753953 10.1189/jlb.1111537 PMC3441312

[137] Rath M , et al., Metabolism via arginase or nitric oxide synthase: two competing arginine pathways in macrophages. Front Immunol. 2014;5:532. 25386178 10.3389/fimmu.2014.00532 PMC4209874

[138] Van den Bossche J , et al., Pivotal advance: arginase‐1‐independent polyamine production stimulates the expression of IL‐4‐induced alternatively activated macrophage markers while inhibiting LPS‐induced expression of inflammatory genes. J Leukoc Biol. 2012;91:685–699. 22416259 10.1189/jlb.0911453

[139] Everts B , et al., TLR‐driven early glycolytic reprogramming via the kinases TBK1‐IKKvarepsilon supports the anabolic demands of dendritic cell activation. Nat Immunol. 2014;15:323–332. 24562310 10.1038/ni.2833 PMC4358322

[140] Wu D , et al., Type 1 interferons induce changes in core metabolism that are critical for immune function. Immunity. 2016;44:1325–1336. 27332732 10.1016/j.immuni.2016.06.006 PMC5695232

[141] Zhao Q , et al., Activated CD69+ T cells foster immune privilege by regulating IDO expression in tumor‐associated macrophages. J Immunol. 2012;188:1117–1124. 22184722 10.4049/jimmunol.1100164

[142] Munn DH , et al., Inhibition of T cell proliferation by macrophage tryptophan catabolism. J Exp Med. 1999;189:1363–1372. 10224276 10.1084/jem.189.9.1363 PMC2193062

[143] Pesce JT , et al., Arginase‐1‐expressing macrophages suppress Th2 cytokine‐driven inflammation and fibrosis. PLoS Pathog. 2009;5:e1000371. 19360123 10.1371/journal.ppat.1000371 PMC2660425

[144] Rodriguez PC , et al., Arginase I production in the tumor microenvironment by mature myeloid cells inhibits T‐cell receptor expression and antigen‐specific T‐cell responses. Cancer Res. 2004;64:5839–5849. 15313928 10.1158/0008-5472.CAN-04-0465

